# Elucidating the importance of the catabolic enzyme, methionine-gamma-lyase, in stresses during Arabidopsis seed development and germination

**DOI:** 10.3389/fpls.2023.1143021

**Published:** 2023-06-06

**Authors:** Yael Hacham, Odelia Shitrit, Ortal Nisimi, Meital Friebach, Rachel Amir

**Affiliations:** ^1^Laboratory of Plant Science, MIGAL – Galilee Research Institute, Kiryat Shmona, Israel; ^2^Tel-Hai College, Faculty of Sciences and Technology, Upper Galilee, Israel

**Keywords:** abiotic stresses, isoleucine, methionine, methionine gamma-lyase, *mgl* mutant, RNAi lines, MGL in seed development/germination

## Abstract

The sulfur-containing essential amino acid, methionine, is a key metabolite in plant cells since it is used as a precursor for the synthesis of vital metabolites. The transcript level of methionine’s catabolic enzyme, methionine γ-lyase (MGL), accumulates in the seeds to a high level compared to other organs. The aim of this study was to reveal the role of MGL during seed development and germination. Using [^13^C]*S*-methylmethionine (SMM), the mobile form of methionine that is used to feed flower stalks of wild-type (WT) plants, revealed that the contents of [^13^C]methionine in seeds were significantly reduced when the plants underwent heat and osmotic stresses. Moreover, the levels of [^13^C]isoleucine, a product of MGL, significantly increased. Also, using the MGL promoter and gene fused to the GUS reporter gene, it was demonstrated that the heat stress significantly increased the protein level in the seeds. Therefore, we can conclude that MGL became active under stresses apparently to produce isoleucine, which is used as an osmoprotectant and an energy source. Transgenic *Arabidopsis thaliana* RNAi seeds with targeted repression of *AtMGL* during the late developmental stages of seeds show that the seeds did not accumulate methionine when they were grown under standard growth conditions, unlike the *mgl*-2, a knockout mutant, which showed a three-fold higher level of methionine. Also, when the RNAi plants developed under mid-heat stress, the level of methionine significantly increased while the content of isoleucine decreased compared to the control seeds, which strengthened the assumption that MGL is active under stress. The germination efficiency of the RNAi lines and *mgl* seeds were similar to their controls. However, the seeds that developed during heat or salt stress showed significantly lower germination efficiency compared to the control seeds. This implies that MGL is important to maintain the ability of the seeds to germinate. The RNAi lines and *mgl* seeds that developed under regular conditions, but germinated during salt or osmotic stress, exhibited a lower germination rate, suggesting an essential role of MGL also during this process. The results of this study show the important role of AtMGL in seeds under stresses.

## 1 Introduction

The levels of the essential amino acid, methionine, are low in plant proteins, thereby limiting the nutritional quality of plant-based diets (Amir, 2008). Methionine also plays a major role in the metabolism of plants since it is used as a precursor to the synthesis of *S*-adenosyl methionine (SAM), which serves as a precursor for phytohormone-ethylene, polyamines, biotin and other essential metabolites (Roje, 2006; Amir, 2010). SAM is also the main donor for the methyl-group in trans-methylation reactions required for DNA, RNA and protein methylation, as well as for the synthesis of many secondary metabolites.

In addition to SAM synthase that produced SAM, two other enzymes catabolized methionine in plants: (i) methionine *S*-methyltransferase (MMT), which uses methionine and SAM to form *S*-adenosyl-homocysteine, and *S*-methyl-methionine (SMM), which serves as methionine storage and a mobile form; and (ii) methionine γ-lyase (MGL) (Amir, 2010).

The current study focuses on the MGL enzyme, whose transcript is strongly regulated by low sulfate (Goyer et al., 2007), exogenous and endogenous methionine (Rébeillé et al., 2006; Goyer et al., 2007; Cohen et al., 2014), osmotic and drought stresses (Less and Galili, 2008; Joshi and Jander, 2009), and the availability of threonine and/or isoleucine (Joshi and Jander, 2009). MGL is a cytosolic enzyme encoded by a single gene (At1g6466) in *Arabidopsis thaliana*, which has a high Km for methionine (~10 mM) (Rébeillé et al., 2006; Goyer et al., 2007). Methionine content does not usually reach such high levels, therefore, the researchers assumed that this enzyme plays additional roles in plant cells (Goyer et al., 2007; Amir, 2010).

The catabolic products of MGL are α-ketobutyrate, ammonia and methanethiol (CH_3_SH) (Rébeillé et al., 2006; Gonda et al., 2010). Methanethiol is a volatile compound that can react with serine to form *S*-methylcysteine, which is a storage molecule for sulfur and methyl groups (Rébeillé et al., 2006; Goyer et al., 2007). It is also proposed that methanethiol can convert to cysteine under sulfur deficiency conditions. Based on this assumption is the observation that the transcript expression of Arabidopsis *MGL* (*AtMGL*) increased 2.5-fold compared to standard conditions under sulfur starvation, suggesting that methionine supports the cysteine content under such conditions (Goyer et al., 2007). The other catabolic product of MGL is α-ketobutyrate, found to be a substrate for isoleucine synthesis. Isoleucine is mainly formed from threonine by threonine deaminase (Rébeillé et al., 2006; Joshi and Jander, 2009; Jander and Joshi, 2010; Joshi et al., 2010). The pathway derived from methionine is considered to be less prominent than threonine deaminase (Rébeillé et al., 2006; Joshi and Jander, 2009; Jander and Joshi, 2010). Isoleucine plays an important role under stresses as an osmoprotectant, but also belongs to six AAs, which, after oxidation, can directly feed electrons into the mitochondrial electron transport chain and thus assist plants in maintaining the energy state of plants undergoing stress (Araújo et al., 2011; Hildebrandt et al., 2015). This role of isoleucine can explain why the transcript expression levels of *AtMGL* are upregulated in leaves during osmotic, drought, salt and other abiotic stresses (Nambara et al., 1998; Less and Galili, 2008; Joshi and Jander, 2009; Avin-Wittenberg et al., 2012; Sham et al., 2015). An indication of the activity of AtMGL under stresses also comes from the observation that the *mgl*-2 knockout mutant shows a significant decrease of isoleucine content in flowers developed under drought stress relative to wild-type (WT) (Rébeillé et al., 2006).

Although the AtMGL protein was detected in all Arabidopsis organs examined, it was not detected in dry mature seeds (Rébeillé et al., 2006). Moreover, the transcript expression level analysis of *AtMGL* showed that its mRNA levels are highly expressed in reproductive tissues, mainly in dry seeds [(Rébeillé et al., 2006; Goyer et al., 2007; Joshi and Jander, 2009); Toronto-Bar website **Supplementary Figure S1**)].

In this study, we aim at gaining more knowledge about the role of AtMGL in *A. thaliana*-developing seeds. We also aim at determining if the protein level of AtMGL is increased in seeds and verifying the role of AtMGL during the early stages of germination. In addition, we aim at revealing if lowering this catabolic enzyme in seeds would lead to higher levels of methionine. If such an increase occurs, it would raise the nutritional quality of the seeds, as previously reported for potatoes (Huang et al., 2014; Kumar and Jander, 2017). In order to address these goals, we used an *mgl* mutant and RNAi::AtMGL lines. The results gave strong indications that AtMGL is active during seed development, mainly when the plant undergoes abiotic stress, and during the early stages of germination, when the seeds undergo stress conditions. Higher levels of methionine in the RNAi lines were detected only under stresses, implying that reducing the level of this enzyme in seeds will not help to increase seed nutritional quality.

## 2 Materials and methods

### 2.1 Generation of transgenic Arabidopsis seeds with reduced *AtMGL* mRNA levels

Arabidopsis (*Arabidopsis thaliana*) plants of ecotype Columbia-0 were used to generate transgenic Arabidopsis seeds with reduced *AtMGL* expression (At1g64660) in a seed-specific manner. Using specific sense and antisense primers (**Supplementary Table S1**), we created fragments with about 300 bp that led to an inverted repeat RNAi cassette. These fragments were amplified using PCR (the list of primers are presented in **Supplementary Table S1**), introduced into a pGEMT plasmid, inserted into an SK-intron intermediate vector (4137 bp), and finally cloned into a pPZP111 binary transformation plasmid containing the seed-specific phaseolin promoter, octopin synthase terminator and NPTII gene for kanamycin selection, as previously described (Cohen et al., 2014). This plasmid was introduced into *Agrobacterium tumefaciens* EHA105 cells, and Arabidopsis transformation was performed by the floral dip method. Transformed seeds were selected on medium supplemented with kanamycin (50 mg mL-1; Duchefa) and carbenicillin (85 mg mL-1; Duchefa). Nineteen independent transformation events were selected and planted in an enriched soil medium. Seeds were collected from the T1 generation and then analyzed for a 3:1 segregation based on the presence of the transgene and the observed resistant phenotype to kanamycin. Segregated T1 lines were then grown again and tested by PCR for the presence of the construct, and the T2 generation was examined for non-segregating lines to produce T2 and further T3 homozygous lines. Transgenic plants having an empty vector containing only the phaseolin promoter and the NPTII gene were generated as a control using the same procedures described above (designated as EV). The knockout *mgl*-2 mutant, which is T-DNA insertion line SALK_103805 (mgl-2; CS16492), was obtained from the Arabidopsis Biological Resource Center (http://www.arabidopsis.org) (Joshi and Jander, 2009).

### 2.2 Generation of transgenic Arabidopsis seeds with AtMGL fused to GUS

The sequence of AtMGL promoter (2250 bp) fused to AtMGL cDNA (including the 5’ UTR) was synthesized with SacII sites at the 5’ and 3’ ends and cloned to the pUC57 vector using GenScript gene synthesis (https://www.genscript.com). The AtMGL fragment was digested with SacII and subcloned into a pKGWFS7 gateway vector (VIB-UGent Center for Plant System Biology) in frame to the GUS reporter gene.

### 2.3 Plant growth and seed collection

The seeds of all tested lines were placed in Petri dishes with 0.5% Murashige and Skoog (MS) medium (Duchefa), 0.5% sucrose (Sigma), and 7 mg mL^-1^ plant agar (Duchefa). The young seedlings were grown for 10 days and then planted on fertilized soil at 22 ± 1°C under a 16/8-h light/dark cycle at a photosynthetic photon flux density of 200 µmol m^-2^ s^-1^, with 50-70% relative humidity. Mature dry seed pools were collected from all genotypes at the end of the desiccation period; each pool comprised at least five different plants. Following collection, the seeds were allowed to dry fully for three days in a vacuum desiccator and stored under dry conditions at 4°C until further analysis.

For the long-term heat and salt stress experiments, plants 42 days after germination (DAG) that contained developing seeds of 12 and 16 (DAF) were used. For the mid-heat stress, the plants were transferred to a growth room with 27±2°C, while for the mid-salt stress, the plants were irrigated with 80 mM of NaCl every four days. The seeds of the tested plants were collected after being dried. For the isotope-labeled and GUS (GUS: β-glucuronidase) experiments, we selected plants 52 DAG that had mainly seeds of 21-26 DAF. The plants were placed at 22°C or at a heat stress of 38°C for 4 h. For abiotic germination assays, 21 seeds from each genotype were grown separately in Petri dishes under normal or stress conditions in four biological replicates (total of 84 seeds). Germination rates (the appearance of radicle) were examined every day and the experiments were repeated twice to observe the same patterns. Salt stress (150 mM NaCl) and osmotic stress (300 mM mannitol) were applied to 0.5% MS medium, while the control for all experiments was 0.5% MS. The materials were manufactured by Sigma-Aldrich.

### 2.4 Transcript analysis using quantitative real-time (qRT-PCR)

qRT-PCR was used to determine the expression levels of *AtMGL* in the different plants examined in this study. Total RNA was extracted using the RNeasy plant mini kit (Qiagen) and RNase-free DNase (Qiagen) according to the manufacturer’s protocol. The reverse transcription reaction was performed using 1 mg of total RNA to synthesize cDNA with the SensiFAST™ cDNA Synthesis Kit (Bioline). To check transcript abundance, gene-specific primers were designed by spanning an intron to reduce the possibility of DNA contamination. The assays were performed in the Corbett Rotor-Gene 6000 real-time PCR system using the qPCRBIO SyGreen Blue qPCR kit (PCR Biosystems Inc.). The conditions were set as follows: an initial polymerase activation step for 2 min at 95°C, followed by 40 cycles at 95°C for 5 sec and 60°C for 25 sec. To normalize variance among samples, we used the constitutive AtPP2A gene as an endogenous control (Czechowski et al., 2005). Ct of the reference gene, AtPP2A, was similar for the samples and repeats (± 0.5 cycle). Efficiency (E) was 0.98 with slope M = -3.37. The list of pairs of primers that were used are presented in **Supplementary Table S1**.

### 2.5 Amino acids profiling using gas chromatography-mass spectrometry (GC-MS)

Mature dry seeds were collected from each plant of the different plant sets. Ten mg of mature dry seeds was treated and derivatized as previously described (Cohen et al., 2016). The single-ion mass method was used for soluble AAs determination with the RXI-5-Sil MS capillary column (RESTEK; 30 m, 0.25-mm i.d. and 0.25-mm thickness). The total-ion-count method was used for metabolic profiling and separation using the VF-5ms capillary column (Agilent; 30 m + 10 m EZ-guard, 0.25-mm i.d. and 0.25-mm thickness). All analyses were carried out on a GC-MS system (Agilent 7890A) coupled with a mass selective detector (Agilent 5975c) and an MPS2 Gerstel multipurpose sampler.

### 2.6 Quantitative GUS activity assay

Seeds of 21-26 DAF were pre-fixed in ice-cold 90% acetone for 10 min (Savaldi-Goldstein et al., 2008). Acetone was removed from the samples by adding washing solution (50 mM sodium phosphate (pH 7.2), 1 mM EDTA, 0.1% Triton x-100, 2.5 mM potassium ferrocyanide, 2.5 mM potassium ferricyanide and 20% methanol) for 10 min. The wash was repeated three times. Samples were placed into staining solution (washing solution with 1.5 mM X-gluc) and incubated at 37°C in the dark for 14 h.  Staining solution was removed and replaced with 70% ethanol for storage. After two days, the seeds were observed under an Olympus SZ61 microscope and photographs were taken with an Olympus SC 180 camera. The intensity of the GUS staining was measured using ImageJ software.

### 2.7 Statistical analyses

Bar graphs were compiled using GraphPad Prism 5.01 scientific software (http://www.graphpad.com/). Significance was calculated using the two-way ANOVA test of *p ≤* 0.05 and examined between the different sets of plants.

## 3 Results

### 3.1 The transcript expression of *AtMGL* and methionine content during seed development and germination

**Figure 1:**
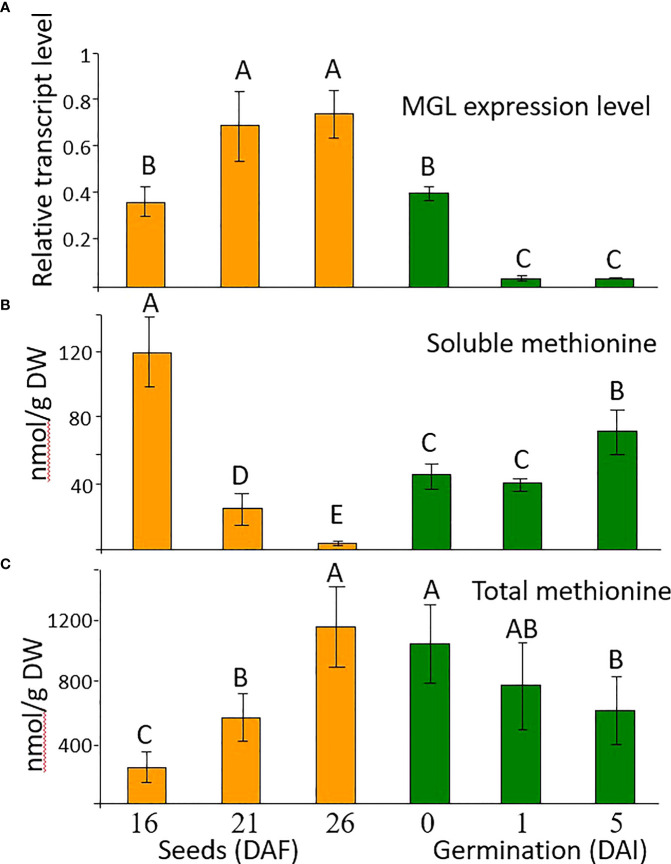
**(A)** The transcript expression level measured by quantitative real-time PCR analysis of *AtMGL* at 16, 21 and 26 days after flowering (DAF), after two days of imbibition in the cold (4 °C) and dark (0), and 1 and 5 days after imbibition (DAI) in light, in wild-type *A thaliana*. The expression data were normalized to the constitutive *AtPP2A* control gene. Data shown are means ± SE of three replicates. **(B)** Soluble methionine contents and **(C)** total methionine content after protein hydrolysis measured using GC-MS analysis. The methionine levels were normalized according to the internal standard of norleucine. Data shown are means ± SE of six replicates. Statistically significant difference (one-way ANOVA, *p*<0.05) is represented by different letters.

Flowers and seeds have elevated accumulation of *AtMGL* mRNA compared to leaves [(Rébeillé et al., 2006; Goyer et al., 2007; Joshi and Jander, 2009); **Supplemental Figure S1**)]. To examine the role of AtMGL in Arabidopsis seeds on methionine level during seed development and the early stage of germination, we examined its transcript and methionine levels. Three stages of seed development – 16, 21 and 26 days after flowering (DAF) – were selected, as well as 48 h after imbibition, and then on the first and the fifth day after placing the seeds on the agar plate. During seed development, the expression level of *AtMGL* significantly increased, while 48 h after the imbibition, the expression level of *AtMGL* decreased and continued to decrease up to the first day after placing the seeds on an agar plate (**Figure 1**). This finding suggests that AtMGL plays a role in the later stages of seed development and during the imbibition, but much less in the latter germination stages.

To get an indication of the relationship between *AtMGL* expression and the level of methionine, the levels of soluble and total methionine that were incorporated into seed proteins were measured at these stages. The dramatic reduction in the soluble level of methionine during seeds development was accompanied by an increased level of total methionine (**Figures 1B, C**). Therefore, we assume that the reduction in soluble level of methionine is mainly due to higher incorporation into seed proteins (Amir et al., 2018). However, we cannot exclude the possibility that the enzymatic activity of AtMGL could also contribute to this reduction. Most probably, the low methionine level at these stages is not due to a reduction in its synthesis, since the expression of cystathionine γ-synthese (*AtCGS*), the key enzyme of methionine synthesis (Chiba et al., 1999; Hacham et al., 2006), was kept at a high level (**Supplementary Figure S1**).

The higher level of total methionine implies that despite the high transcript expression of *AtMGL* during seed development, AtMGL is not significantly active in the seeds. These findings are in accordance with Rébeillé et al. (2006), who did not detect the protein level of AtMGL in the seeds, unlike in other organs.

### 3.2 Producing transgenic seeds expressing the RNAi::AtMGL

**Figure 2:**
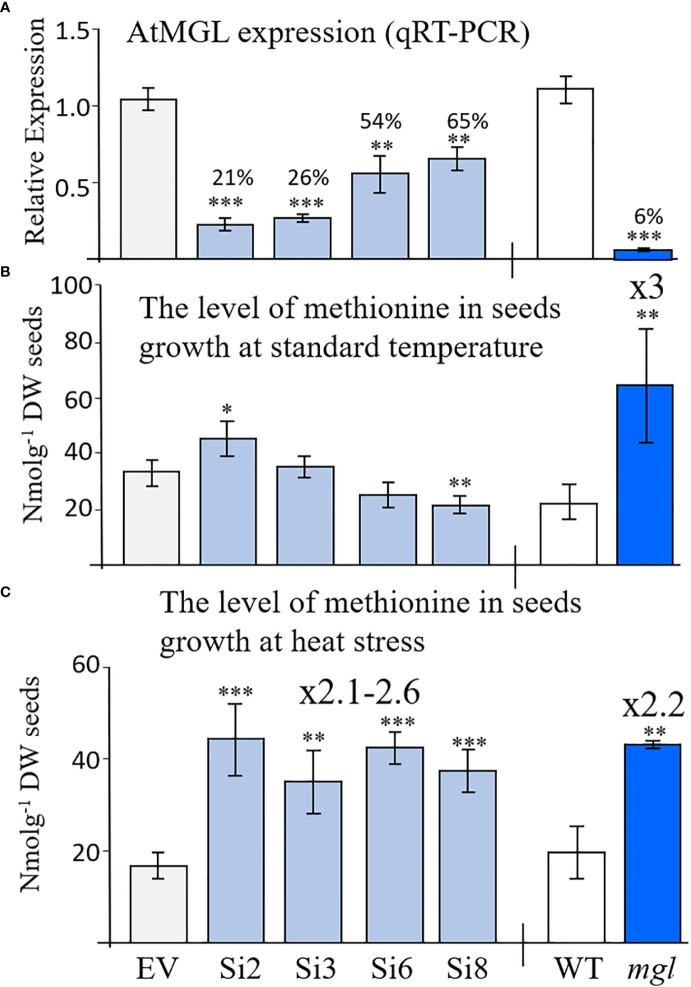
**(A)** Relative transcript expression level of *AtMGL* measured by quantitative real-time PCR analysis in dry mature seeds homozygous RNAi (Si) lines compared to the empty vector (EV) (left panel), wild-type (WT) and *mgl* mutant (right panel). The expression data were normalized to the constitutive *AtPP2A* control gene. **(B)** The content of methionine in seeds grown at regular temperature (22-24°C) or under **(C)** heat stress (27°C). The data are presented as the mean ± SD obtained from four independent measurements. Statistically significant changes using Student’s T-test are identified by an asterisk *(*p*<0.05), **(*p*<0.01), ***(*p*<0.001) between the RNAi lines and *mgl* to their controls, EV and WT, respectively.

In order to assess the role of AtMGL in methionine content during seed development and germination, we generated transgenic Arabidopsis seeds expressing RNAi::AtMGL under the control of the seed-specific promoter of phaseolin (**Supplementary Figure S2A**). As a control, we generated a plasmid carrying solely this promoter, called empty vector (EV) (**Supplementary Figure S2A**). The phaseolin promoter is the abundant seed-storage-protein in the common bean (*Phaseolus vulgaris*) and is turned off during the vegetative stages of plant development (Li and Hall, 1999). It was also demonstrated that this promoter is induced constitutively during the maturation and desiccation stages of Arabidopsis seed development (Fait et al., 2011) when the transcript of the *AtMGL* accumulates (**Figure 1**; **Supplementary Figure S1**). Seeds from 19 kanamycin-resistant plants were screened by quantitative real-time PCR (qRT-PCR), and four transgenic lines, Si2, Si3, Si6 and Si8, exhibiting the lowest expression levels of *AtMGL* transgene (**Supplementary Figure S2B**) were selected for further growth and analysis. The morphology and growth rates of these transgenic plants were similar to those of the EV. The expression of *AtMGL* was examined in the seeds of the T3 homozygous plants to show 21%, 26%, 54% and 65% expression of the EV (**Figure 2A**).

### 3.3 The *AtMGL* transcript expression level in the homozygous mutant of *mgl*

To gain a deeper understanding of the importance of this gene in seeds, we also examined the *mgl*-2 mutant (*mgl-2* SALK_103805), which has a T-DNA insertion in the first exon (Joshi and Jander, 2009). The transcript expression level of *AtMGL* in seeds was measured after genotyping and the production of homozygous plants. The expression level of *AtMGL* in the seeds of this mutant was 6.3% from the WT (**Figure 2A**). The mutant does not have visible phenotypes or growth defects, as previously reported (Goyer et al., 2007; Joshi and Jander, 2009).

### 3.4 The level of methionine in transgenic seeds having RNAi::AtMGL and in the *mgl* mutant

If the enzyme of AtMGL is active in seeds, a lower expression level of *AtMGL* in the RNAi seeds should lead to a higher methionine content and a lower level of isoleucine compared to the EV. To confirm this assumption, the levels of soluble methionine, isoleucine and other AAs were examined in the dry seeds of the four selected RNAi lines using GC-MS. The results showed that the level of methionine significantly increased in line Si2 by 24% compared to the EV, did not change significantly in lines Si3/Si6, and significantly decreased in the Si8 line (**Figure 2B**; **Table 1**). The level of isoleucine did not change significantly in the RNAi lines. This suggests that the activity of AtMGL is low in the developing seeds.

**Table 1:** Soluble amino acid contents in dry seeds of the EV, four seed-specific lines expressing the RNAi::AtMGL (A); wild-type (WT) and the *mgl* mutant (B).

	(A)						(B)	
AAs	EV	Si2	Si3	Si6	Si8		WT	*mgl*
Alanine	21.6 ± 5	19.2 ± 2.1	20 ± 1.1	21.8 ± 0.8	35.6 ± 11.7	92.9 ± 14	25.4 ± 4.5 *
Valine	3.6 ± 2.7	8.2 ± 4.7	17.2 ± 7.7	22.6 ± 3.4 *	33.1 ± 7.1 *	60.6 ± 76.3	39.9 ± 36.5
Serine	58.3 ± 2.2	51.9 ± 12.1	44.6 ± 5.7 *	2.4 ± 0.3 *	3.9 ± 0.9 *	125.1 ± 0.2	100.1 ± 14.5
Leucine	13.7 ± 5.3	21.6 ± 9.2	40.1 ± 8.9 *	26.5 ± 11.8	25.8 ± 4.4 *	23.5 ± 13.7	30.8 ± 19.3
Threonine	44.6 ± 5.6	48.4 ± 3.3	48.4 ± 3.3	7.4 ± 0.6 *	9.8 ± 3.9 *	67.1 ± 16.3	64.6 ± 18.4
Isoleucine	5 ± 1.2	7.2 ± 1.4	7.6 ± 1.8	2.1 ± 0.9	3.2 ± 1	34.8 ± 1.4	12.6 ± 4.2 *
Glycine	42.7 ± 5	56.9 ± 10	31.9 ± 9.4	10.8 ± 1.3 *	12 ± 1.8 *	66.2 ± 21.7	229.5 ± 83.7 *
Homoserine	7.8 ± 1.2	11.2 ± 1.9 *	11.4 ± 2.7	20.9 ± 4.4 *	25.5 ± 5.4 *	9 ± 0.6	14.6 ± 0.7 *
Methionine	18.4 ± 2.4	25.2 ± 3.4 *	19.5 ± 1.7	13.9 ± 4.1	11.8 ± 1.3 *	13 ± 2.4	39 ± 8.5 *
Aspartate	259 ± 37.6	302.1 ± 75.6	248.4 ± 18.3	217.6 ± 56.2	212.1 ± 19.4 *	153.8 ± 49.2	272.8 ± 7.4 *
Phenylalanine	54.1 ± 2.3	89.3 ± 17.3 *	52.1 ± 1.3	54.3 ± 6.5	44.4 ± 4.6	58.4 ± 13.1	63.7 ± 8.2
Glutamate	111.5 ± 35.9	104 ± 25.8	97.4 ± 19.9	82.3 ± 24.6	95.1 ± 43.7	405 ± 181.7	106 ± 34.3
Homocysteine	9.8 ± 1.6	10.2 ± 1.7	9.8 ± 0.7	9.5 ± 1.5	9.9 ± 2	13.2 ± 3.1	10.4 ± 1.2
Asparagine	54.8 ± 5.4	62.9 ± 9.4	55.4 ± 7	50 ± 5.4	45.9 ± 3.8 *	71.1 ± 12.1	70.2 ± 3.9
Tyrosine	53.5 ± 6.6	75.8 ± 5.2 *	62.1 ± 12.8	94.4 ± 9.3 *	74.7 ± 7.7 *	56.7 ± 20.8	68.4 ± 1.4

The content was examined in dry seeds using GC-MS.*

*The amounts of amino acids were calculated based on norleucine that was used as an internal standard. The results are mean ± SD of five replicates. Student’s T-test was used to determine statistically significant differences (p<0.05) between the EV and each of the RNAi lines, as identified by an asterisk. The seeds of WT and mgl were examined at a different time from the other seeds of the RNAi lines/EV.

Contrary to these results are those obtained from the *mgl* mutant that grown under the same conditions. This mutant, which lacks MGL activity (Joshi and Jander, 2009), had a 3-fold higher level of methionine compared to the WT seeds [as previously reported (Joshi and Jander, 2009)], while the level of isoleucine decreased by 2.7-fold (**Figure 2B**; **Table 1**). The higher level of methionine in the mutant might result from a lowered activity of AtMGL. It could also result from higher transport of methionine from non-seeds tissues. The levels of the other AAs (**Table 1**) showed that several AAs significantly increased in the seeds, while others significantly decreased in some of the examined lines, but not in a stable way in all the four RNAi lines (**Table 1A**). In the *mgl*, significant lower level of alanine, was detected, while the levels of glycine, homoserine and aspartate were significantly higher than WT (**Table 1B**). Together, the finding implies that reducing the *MGL* using the seed-specific RNAi method does not contribute to higher methionine content, an aspect that is important from a biotechnological point of view.

### 3.5 Initial evidence of higher activity of AtMGL in seeds that developed under heat stress

The results obtained from the RNAi lines basically suggest that under normal conditions, there is low activity of the MGL enzyme inside the seeds, therefore partially lowering its level does not significantly affect methionine levels. Thus, at this stage, we searched for the conditions leading to a higher activity of the MGL enzyme. Previous studies (Less and Galili, 2008; Joshi and Jander, 2009) and data obtained from the Toronto-Bar analysis (http://bar.utoronto.ca/; **Supplementary Figure S3**) suggest that the transcript expression level in leaves significantly increases under abiotic stresses. These data suggest that under those conditions, the enzyme becomes active. To examine this possibility in seeds, the different sets of plants were transferred to a growth room whose temperature increased to 27±2°C (about 5°C higher than under regular conditions). The transfer occurred when the first developing seeds were more than 12 DAF, the time that the phaseolin promoter began to be active (Fait et al., 2011). The level of methionine was determined in the dry seeds of these plants. The results showed that the methionine levels were significantly higher (2.1- to 2.6-fold) in the RNAi lines compared to the EV (**Figure 2C**; **Table 2A**), while the levels of isoleucine were lower than the EV (2.3- and 3.3-fold) (**Table 2**).

**Table 2:** Soluble amino acid contents in dry seeds of the EV, four seed-specific lines expressing the RNAi::AtMGL (A); wild-type (WT) and the *mgl* mutant (B). wild-type (WT) and the *mgl* mutant.

	(A)						(B)	
AAs	EV	Si2	Si3	Si6	Si8		WT	*mgl*
Alanine	94.5 ± 8	83.9 ± 4.1	71.7 ± 7.4 *	67.3 ± 2.9 *	65.1 ± 0.01 *	94.2 ± 23.8	71.9 ± 10.1
Valine	58.7 ± 7.9	45.3 ± 9.8	21.9 ± 9.8 *	24.9 ± 7.9 *	22.2 ± 0.01 *	62.3 ± 41.4	55.6 ± 32.3
Serine	93.4 ± 6.2	71.5 ± 6 *	64.3 ± 11.7 *	66 ± 4.7 *	66.1 ± 0.01 *	99 ± 65	111 ± 114.8
Leucine	22.6 ± 4.4	13.7 ± 2.2 *	9.2 ± 0.8 *	7.4 ± 0.4 *	8.6 ± 0.003 *	30.9 ± 15.6	23.1 ± 15.4
Threonine	55.4 ± 11.9	53.9 ± 6.2	53.9 ± 6.2	44.7 ± 7.1	32.2 ± 0.02 *	84.2 ± 25.4	109.5 ± 40.5
Isoleucine	24.5 ± 4.5	10.6 ± 2.8 *	7.3 ± 1.4 *	7.9 ± 0.9 *	7 ± 0.01 *	37.6 ± 6.8	6.9 ± 2.1
Glycine	67.8 ± 3.9	45.6 ± 8.5 *	45 ± 9.6*	40.5 ± 5.7 *	54.9 ± 6.4*	63.2 ± 30.2	70.5 ± 47.9
Homoserine	9 ± 3.2	9.2 ± 0.9	7.8 ± 1	6.9 ± 1.3	6.3 ± 0.02	9.2 ± 6.3	14.1 ± 4.1
Methionine	11.7 ± 2.5	30.7 ± 5.3 *	24.2 ± 5.2 *	29.7 ± 2.1 *	25.9 ± 0.001 *	13.5 ± 3.9	22.3 ± 0.3 *
Aspartate	153.3 ± 35.2	79.1 ± 13.8 *	67.4 ± 11 *	65.3 ± 2.6 *	63.1 ± 0.002 *	153.8 ± 121.8	73.1 ± 67.3
Phenylalanine	59.2 ± 8	68.8 ± 4.3	65.3 ± 4.9	86.3 ± 4.2 *	67.3 ± 0.4	59.3 ± 13.3	60.5 ± 18.7
Glutamate	404.1 ± 59.5	345.1 ± 87.8	212.4 ± 33.2 *	260.2 ± 47.4 *	263.4 ± 11.5 *	405 ± 101.6	348.3 ± 77.4
Homocysteine	13.2 ± 1.1	12.8 ± 1.1	12.5 ± 1.1	13.6 ± 2.6	13.3 ± 2.1	13.1 ± 2.7	11.9 ± 1.1
Asparagine	161.5 ± 19.4	151.8 ± 28	118.7 ± 23.4	176 ± 22.8	139.5 ± 72.9	72 ± 4.3	62.7 ± 4.5 *
Tyrosine	58.1 ± 6.3	83.6 ± 14.8 *	83.1 ± 8.7 *	100.8 ± 5.3 *	102 ± 14 *	709 ± 32.6	731.9 ± 62

The seeds were developed on plants undergoing slight heat stress of 27°C. The content was examined in dry seeds using GC-MS.*

*The amounts of amino acids were calculated based on norleucine that was used as an internal standard. The results are mean ± SD of five replicates. Student’s T-test was used to determine statistically significant differences (p<0.05) between the EV and each of the RNAi lines, as identified by an asterisk. The seeds of WT and mgl were examined at a different time from the other seeds of the RNAi lines/EV.

Next, we measured the level of methionine in the WT and *mgl*. The level of methionine increased by a similar content of 2.2-fold to those developed under non-stress conditions, while the level of isoleucine significantly decreased by 5.4-fold (**Table 2A**). Upon examining the levels of the other AAs in the RNAi lines, it was found that the levels of five other AAs, serine, valine, leucine, aspartate and glutamate, significantly decreased, while only the level of tyrosine significantly increased (**Table 2A**). The determination of AAs in *mgl* showed that the level of asparagine significantly decreased (**Table 2B**).

Two possibilities could lead to a higher level of methionine in the RNAi lines compared to the EV. The first is less catabolism of methionine due to the lower expression level of *AtMGL*; the second is that the expression level of *AtCGS*, the key enzyme of methionine synthesis, increased in these lines. To determine that AtCGS is not involved in this case, a qRT-PCR analysis was performed showing that its level decreased significantly by 73% to 89% compared to the EV in the RNAi lines (**Supplementary Figure S4**), suggesting that this enzyme is not responsible for the higher level of methionine in these lines. The reduction of *AtCGS* might result from the higher level of methionine whose feedback regulates its transcript level (Chiba et al., 1999). We also determined using this analysis that the transcript level of *AtMGL* did not change in the seeds under stress. Together, the results suggest that under heat stress, the AtMGL became more active and increased methionine content in the RNAi lines.

Due to these changes, we also studied the morphological phenotypes of the RNAi lines. No significant differences were observed between the different sets of plants. To detect the changes occurring in seeds, 200 seeds from the RNAi lines and the EV that had undergone heat stress were examined. The seeds of the RNAi exhibited similar morphology and dimensions to the EV. Their total protein and reducing sugars that represent starch content were also examined to reveal that the level of these major storage components were not significantly changed compared to the EV. This, in some way, was unexpected since under stresses (mainly abiotic), proteins degrade and some of the AAs can be used as an alternative source of substrate to form energy (Araújo et al., 2011; Hildebrandt et al., 2015; Galili et al., 2016). The observation that the levels of the storage compounds did not decrease could be because the stress was not great and the plants and their seeds were able to cope with it.

### 3.6 Feeding analysis strengthens the assumption that AtMGL is active under stresses inside seeds

**Figure 3:**
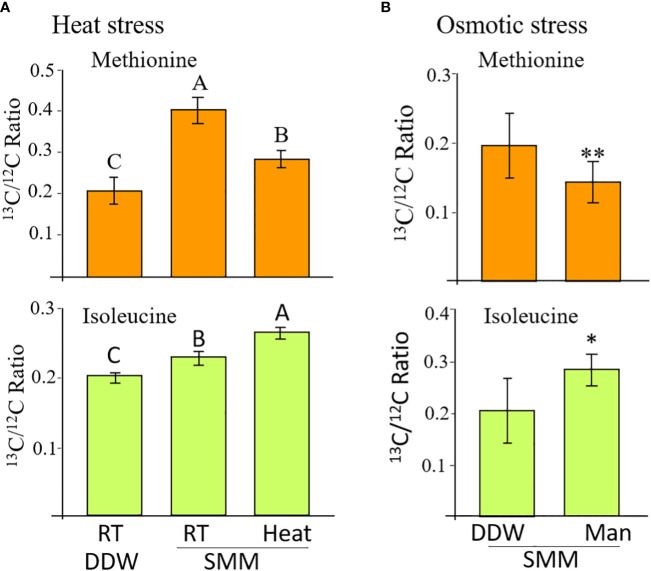
The levels of labelled methionine and isoleucine in WT seeds whose flower stalks were exposed to heat stress **(A)** and osmotic stress **(B)**. The control treatment for the heat stress was flower stalks placed at room temperature (RT 23°C) in double-distilled water (DDW) or 2 mM of [^13^C]SMM (RT SMM). For the heat stress, the flower stalks were exposed to 39°C for 16 hours. For the osmotic stress, the flower stalks were exposed to 300 mM of mannose (Man) for 8 hours. The data are presented as the ratio between the isotopes labeled to unlabeled methionine or isoleucine. The results are mean ± SD of four independent measurements. For the heat stress, statistically significant differences (one-way ANOVA, *p*<0.05) are represented by letters. For the osmotic stress, statistically significant differences using the independent Student’s T-test are identified by an asterisk *(*p*<0.05), **(*p*<0.01) between those undergoing stress (Man) and control non-stress conditions (DDW).

Our efforts to form antibodies against AtMGL that could help us determine the protein level of MGL in seeds basically failed. We also did not succeed in obtaining reliable results from the enzymatic activity of MGL. To overcome these failures, we decided to use ^13^C-labeled methionine and test the labeled isoleucine that formed from methionine as a marker for MGL activity. This could also determine a direct link between these two AAs since isoleucine is usually formed from threonine by threonine deaminase (Joshi and Jander, 2009; Joshi et al., 2010). It was previously shown that methionine transfers in plants from one organ to the other in the form of SMM (Kocsis et al., 2003; Cohen et al., 2017). Thus, we used the [^13^C]methionine to form [^13^C]SMM [according to (Cohen et al., 2017)]. To obtain uniformity of results, the inflorescent flower stalks of WT plants were removed from the rosette leaves and placed in a tube with distilled water (as a control) or in water with [^13^C]SMM (2 mM). Half of the tubes were placed in an incubator at a temperature of 23 °C and half in an incubator at 39 °C. After 16 hours, the siliques were harvested to liquid nitrogen and the seeds were separated. The levels of labeled and unlabeled methionine and isoleucine were quantified using GC-MS, and the ratio was calculated (**Figure 3A**).

The labeled methionine levels in the seeds of inflorescent flower stalks placed in [^13^C]SMM showed a significant increase of 1.9 times compared to those placed in water, indicating that [^13^C]SMM was transferred to the seeds and converted there to [^13^C]methionine, as we showed previously (Cohen et al., 2017). To check if heat stress affects MGL activity, the level of labeled methionine and isoleucine were compared between seeds undergoing heat stress and those maintained under 23°C. Under heat stress, the level of [^13^C]methionine decreased significantly by 43%, while the level of [^13^C]isoleucine increased significant by 22% compared to the control seeds (**Figure 3A**). The levels of threonine and valine did not change significantly in the seeds of the different sets (data not shown).

In addition to heat stress, the effect of 300 mM of mannitol causing osmotic stress was tested (**Supplementary Figure S5**). The application of the SMM to the inflorescent flower stalks was done in the same way as described for the heat stress. The tubes were placed in an incubator at 23 °C and the seeds were collected after 8 hours. A significant 40% decrease in the [^13^C]methionine level was detected in the seeds of the mannitol treatment compared to the control treatment. The level of [^13^C]isoleucine increased significant by 37% in the mannitol-treated seeds (**Figure 3B**). The results from these two experiments strongly suggest that AtMGL is active under stresses inside the WT seeds, and methionine is used to produce isoleucine in seeds under these conditions.

### 3.7 Expression of AtMGL in seeds using the GUS reporter gene

**Figure 4:**
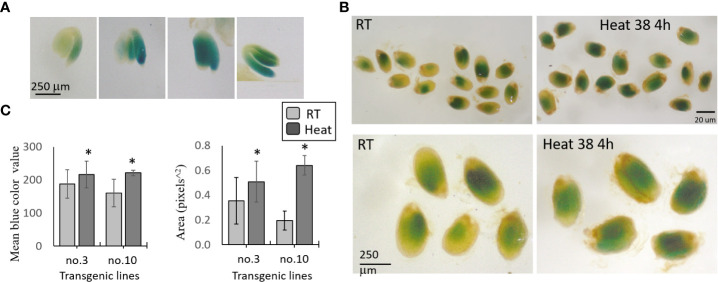
GUS staining of transgenic seeds expressing the AtMGL-promoter-cDNA of AtMGL:: GUS. The seeds are stained with X-Gluc for 24 h. **(A)** GUS activity in the embryo, root and cotyledons of four representative seeds. **(B)** Representatives of seeds exposed to room temperature (RT, 22°C) or heat at 38°C for 4 hours. Seeds that represent the seeds at RT and under heat stress (upper panel), and their magnification (lower panel). **(C)** The mean of the blue color value, and the area in 40 seeds to each of two transgenic lines (no. 3 and no. 10). Significant differences using the Student’s T-test are identified by an asterisk *(*p ≤* 0.05) between those undergoing heat stress and control non-stress conditions.

In order to determine whether the protein level of AtMGL is increased in the last stages of seed development, and to strengthen the assumption that its level increases in seeds during abiotic stresses, we produced a new binary construct. This construct contain 2000 bp of the promoter and the cDNA gene of AtMGL, which fused to the GUS (β-glucuronidase)-reporter gene. The construct was used to transform WT Arabidopsis plants. Ten lines were selected based on their resistance to kanamycin. All of these lines exhibited GUS activity in their developing seeds (21-26 DAF), albeit at different intensities. The results gave a strong indication that the protein of AtMGL was increased in the seeds during the late stage of seed development. GUS staining was detected only in the embryo, mainly in the root and cotyledons (**Figure 4A**).

To determine if the protein of MGL increased under stresses, we selected two transgenic lines that showed a relatively high content of GUS (no. 3 and no. 10). The plants were exposed to 22°C and 39°C for 4 h. The results taken from about 40 seeds in each treatment and the control showed that the mean gray value (representing the intensity of the blue color by pixels) and the GUS detected area significantly increased under heat stress in the two lines compared to the control, which was not exposed to stress (**Figures 4B, C**). Together, the results show that heat stress significantly increased the protein content of AtMGL in the embryo inside the seeds.

### 3.8 The effect of heat stress that occurred during seed development on germination efficiency

**Figure 5:**
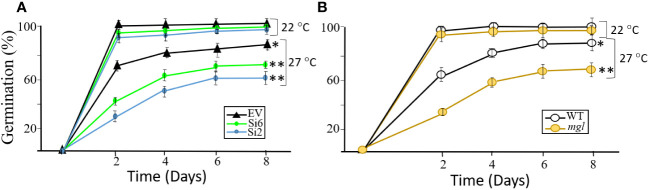
The effect of heat stress (27°C) occurring during seed development on germination efficiency and germination rate of the seeds. The control plants were grown under 22°C. **(A)** The effect on seeds belonging to the EV and two RNAi lines (Si2 and Si 6). **(B)** The effect on seeds of the WT and the *mgl* mutant. The data mean represent four agar plates, in which each plate had 21 seeds, a total of 84 seeds ± SD. Significant differences using the Student’s T-test are identified by an asterisk *(*p ≤* 0.05); **(*p*<0.01) between those undergoing stress and control non-stress conditions.

Since the transcript expression levels of the *AtMGL* increased with seed development (**Figure 1**), it was assumed that this enzyme is also important for the first stages of seed germination (Rébeillé et al., 2006). At this stage, the seed-storage proteins degrade and the AAs are used to form new proteins, to synthesize other metabolites and to serve as a source of energy (Hildebrandt et al., 2015; Galili et al., 2016). If this is the case, it is expected that lowering the expression levels of *AtMGL* in the RNAi lines that cope with stress during development will reduced germination efficiency and slower the germination rate. For this experiment, we selected the RNAi lines of Si2 and Si6 that had the lowest transcript expression levels of *AtMGL*, as well as the WT and *mgl*. To test this assumption, we examined the germination rate and efficiency of the seeds that developed under standard conditions, and those that developed under heat stress. The seeds were placed on regular agar medium (0.5 MS) show that the seeds of the RNAi lines that developed under non-stress conditions germinated at a similar rate and efficiency as the EV, and reached almost 100% germination already on the second day (**Figure 5A**). Similar results were obtained for the WT and *mgl* (**Figure 5B**). However, the seeds of all of the lines that developed under heat stress showed a significantly lower germination capacity and delay in germination rate. For the EV, the germination efficiency decreased to 86%, while that of the RNAi lines significantly reduced to 72% to 60% (**Figure 5A**). The germination efficiency of the WT seeds and *mgl* mutant was reduced to 81% and 60%, respectively (**Figure 5B**).

Together, the results suggest that seeds of RNAi and *mgl* plants that developed under regular growth conditions, germinate well, suggesting the MGL activity at such condition is not essential for the germination processes. However, when the seeds developed under stress conditions, AtMGL plays an essential role enabling the seeds to develop properly and prepare them to germinate well.

### 3.9 The effect of exposure to salt stress during seed development on the ability of seeds to germinate

In order to strength the assumption that stress that occurs during seed development damages seeds having lower levels of MGL, we carried out an additional experiment. To this end, the plants were grown under slight salt stress, which they were irrigated every four days with 80 mM of NaCl when the first developing seeds were more than 12 DAF. This NaCl concentration was selected since it is not toxic and enables normal growth of the plants (**Supplementary Figure S6**). As a control, the plants were irrigated with water. The irrigation ended when the seeds were dry, and the dried seeds were used for analysis. To determine how moderate salt stress affected these plants, we measured the total yield of the seeds. In all of the lines, the salt treatment lowered the yield compared to the untreated control plants. The yield was reduced in the EV and WT by about 5% and 8%, respectively, while the Si2 and Si6 RNAi lines had significantly lower yield of 17% and 21%, respectively, compared to the EV. A reduction was also detected in the seeds of the *mgl* mutant, which showed a significantly lower yield of 55% compared to the untreated plants and its control, WT. The seed weights of 200 seeds from the all of the tested lines showed a reduction of 4-8% compared to the control seeds that were grown under regular conditions.

**Figure 6:**
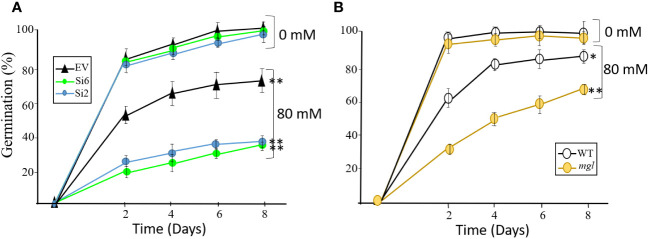
The effect of salt stress (80 mM) occurring during seed development on germination efficiency and germination rate of the seeds. The control plants were grown in water. **(A)** The effect on seeds belonging to the EV and two RNAi lines (Si2 and Si 6). **(B)** The effect on seeds of the WT and the *mgl* mutant. The data mean represent four agar plates, in which each plate had 21 seeds, a total of 84 seeds ± SD. Significant differences using the Student’s T-test are identified by an asterisk *(*p*≤ 0.05; **(*p*<0.01) between those undergoing stress and control non-stress conditions.

The seeds of all lines were placed on regular agar medium to show, as detected for the heat stress, that the seeds of the RNAi lines and the *mgl* that grew without stress germinated at a similar rate and efficiency as their controls, EV and WT (**Figure 6**). However, the seeds of all lines that developed under salt stress showed a significant delay in their germination capacity and rate compared to their controls, seeds that had developed under non-stress conditions. The germination efficiency of the EV decreased from 98% in the control on the eighth day to 71%. However, the germination efficiency of the RNAi lines was significantly lower and reduced to 35-38% (**Figure 6**). In addition, the germination rate of the seeds of the WT and *mgl* decreased from 96-97% in the controls to 87% and 64%, respectively, in those coping with the stress.

Together, the results indicate that when the seeds cope with stress, AtMGL plays an essential role during seed development. Its functions enable the seeds to develop properly and also prepare them to germinate well. The observation that the mutant is less sensitive to stress compared to the seeds of the RNAi lines suggests that is more adapted to salt stress during seed development.

### 3.10 The germination rate of seeds under salt and osmotic stresses

**Figure 7:**
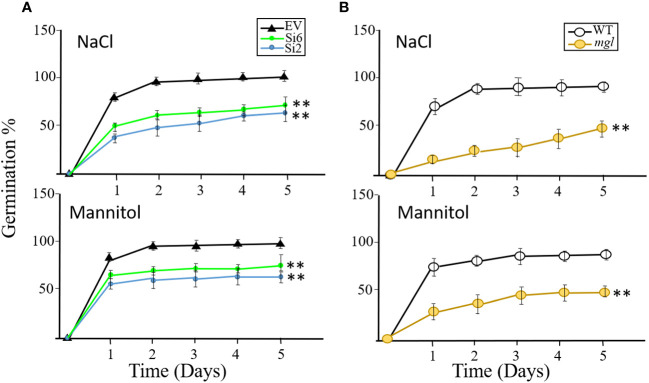
The effect of salt stress (NaCl 150 mM) or osmotic stress (300 mM mannitol) inserted into MS agar medium on germination efficiency and germination rate of the seeds. The seeds were collected from plants that were not exposed to stress when the seeds developed. **(A)** The effect on seeds belonging to the EV and to two RNAi lines (Si2 and Si 6). **(B)** The effect on seeds of the WT and *mgl* mutant. The data mean represent four agar plates, in which each plate had 21 seeds, a total of 84 seeds ± SD. Significant differences using Student’s T-test are identified by an asterisk **(*p*<0.01) between those undergoing stress and control non-stress conditions.

The results described above suggest that seeds of the RNAi lines and the mutant germinate similarly to the control seeds of the EV and WT when they are grown under regular conditions. Next, we were interested to discover if the RNAi and *mgl* seeds that had developed under regular conditions would germinate well when they coped with abiotic stresses such as salt or osmotic stresses during the germination processes. If the transcript *AtMGL* that accumulated in the seeds [**Figure 1**; (Rébeillé et al., 2006)] is required for germination, the rate of the RNAi lines and the mutant should be lower than their controls, EV and WT. To verify this, the seeds of the EV and two RNAi lines, Si2 and Si6, were placed on 0.5 MS medium that contained 150 mM salt or 300 mM mannitol that caused osmotic stress. The germination efficiency of the EV reached almost 92% after the second day, but that of the two RNAi lines showed significantly lower rates, and their germination efficiency was 61-65% under salt stress and 64-72% under osmotic stress. Similar results were obtained for the *mgl* and WT seeds (**Figure 7**). The WT showed lower germination efficiency under these stresses, which, after five days, reached 91%, while the *mgl* reached 42% when they germinated on salt and 43% on mannitol. This suggests that seeds having lower levels of AtMGL had difficulties in germinating under stress conditions, implying that AtGML plays an essential role during germination when the seeds undergo stress.

## 4 Discussion

The results of the current study strongly indicate that: (i) the activity of AtMGL inside the seeds increased when the plants coped with abiotic stresses; (ii) the protein of AtGML was produced in the embryo during seed development; and (iii) seeds with a lower expression level of *AtMGL* had difficulties in germinating when the seeds underwent stress. These points are discussed below.

### 4.1 The activity of AtMGL inside the seeds increased when the plants coped with abiotic stresses

The levels of methionine and isoleucine under standard conditions were not significantly altered in the RNAi lines compared to EV. This is unlike the *mgl* mutant, which has a higher level of methionine but a lower level of isoleucine. These differences between the RNAi lines and the mutant are most likely due to the fact that in the RNAi lines, the AtMGL decreases only in the seeds, while in the mutant, this gene is silenced throughout all of the plant’s organs. It is possible that a high level of methionine in the seeds of the mutant originates from the vegetative tissues that transported the excess methionine to the developing seeds. This assumption must be further tested. The elevation of methionine could also occur due to significantly lower levels of AtMGL in the seeds of the mutant compared to the RNAi lines (**Figure 2**). If this is the case, it suggests that some activity of AtMGL occurs in the WT, and lowering this activity in the mutant leads to higher methionine in the seeds of the mutant. However, in the RNAi lines developed in heat stress and in *mgl*, the level of methionine increased while the level of isoleucine decreased. This gave a first indication that AtMGL is more active in seeds during stresses. This assumption is further strengthened by the data obtained from plants expressing the MGL-GUS and from the labeled SMM experiment. Labeled methionine was previously used to identify the methionine catabolic products SAM, *S*-methylmethionine (SMM) and isoleucine (Rébeillé et al., 2006). Also, using [^13^C5 ^15^N]methionine-fed flower stalks of 30-day-old plants of WT and *mgl*-2 for 24 h showed that the siliques and flowers had high levels of [^13^C4]isoleucine in the WT (Joshi and Jander, 2009). These two studies that were carried out in non-seed tissues did not examine the labeled methionine and isoleucine when those plants underwent stress.

Two main reasons could eventually lead to the need to break down methionine in seeds under stresses. The first is that a high level of methionine in seeds can be toxic. The level of methionine increases in stress together with other AAs and can reach high levels (Nambara et al., 1998; Rosental et al., 2016). Support of this option is evidence that raising methionine in seeds by seed-specific expressing *AtCGS* increased the expression levels of many stress-associated genes and metabolites (Cohen et al., 2014; Cohen et al., 2016; Cohen et al., 2017). These studies suggest that methionine levels are strongly controlled and that excessive methionine is deleterious to seed health. Also, it was suggested that soybean seeds that have a low expression level of *MGL*, hyper-accumulate SMM to avoid the harmful effects caused by excess-free methionine (Teshima et al., 2020). SMM has been considered to be the most mobile storage form of methionine and is a much less toxic compound (Kocsis et al., 2003). The second reason for the need to break down methionine is related to MGL products, especially isoleucine. Isoleucine accumulates as an osmoprotectant under conditions of dryness and osmotic stresses. It can be used to neutralize types of reactive oxygen and as a substrate for the synthesis of proteins associated with stresses (Nambara et al., 1998; Joshi et al., 2010). In addition, isoleucine is used as an alternative pathway to maintain the energy state of the cells under stresses (Araújo et al., 2011; Hildebrandt et al., 2015; Galili et al., 2016).

Apparently due to the role of isoleucine in stresses, the activity of AtMGL became very important under such conditions. Indeed, the transcript expression level of *MGL* upregulated in leaves in response to abiotic stresses, mainly during osmotic, drought and salt stresses (Less and Galili, 2008; Joshi and Jander, 2009; Galili et al., 2014). Also, *AtMGL* belongs to genes whose expression is related to cellular energy production as a response to stress (Less and Galili, 2008; Avin-Wittenberg et al., 2012). The researchers suggest that the high level of MGL under stresses is related to the ability of isoleucine to be used as source of energy since plants under stress must divert energy and resources to cope with the stress response (Zhang et al., 2020).

Since *AtMGL* is upregulated under stress, it was expected that overexpression or downregulation of this gene would affect plants that cope with stress. However, plants having 35S::AtMGL and *mgl* did not show a phenotype of drought tolerance or higher sensitivity, respectively (Atkinson et al., 2013). These observations are likely to result from redundancy with threonine deaminase, which is considered to be the main enzyme that forms isoleucine (Joshi and Jander, 2009; Joshi et al., 2010). These researchers indicate that AtMGL is regulated according to the needs of isoleucine, but its level is subordinate to threonine deaminase. Indeed, *MGL* expression was significantly reduced in flowers of the isoleucine feedback-insensitive threonine deaminase mutant background, which has 20-fold higher isoleucine levels (Mourad and King, 1995). However, this low *MGL* expression increased three-fold during drought stress (Joshi and Jander, 2009). When dehydrated flowers were compared to fresh flowers, the transcript expression of threonine deaminase significantly increased WT and *mgl*-2, suggesting regulation in response to the increased need for isoleucine biosynthesis (Joshi and Jander, 2009).

The involvement of MGL in stresses was also reported in other studies. The expression level of *AtMGL* was upregulated in roots during water deficit, nematode stress and a combination thereof (Atkinson et al., 2013). Moreover, significantly fewer nematodes were observed on 35S::AtMGL plants, which had only 24% the infection rate of WT plants. The researchers suggest that the depletion of methionine may inhibit nematode protein synthesis and thus prevent their development (Atkinson et al., 2013). The transcript expression of *AtMGL* was also found to increase when plants were exposed to a combination of *Butritis cinerea* infection stress with salinity or osmotic stress (Sham et al., 2015).

Taken together, the data obtained on this study show that methionine is degraded to form isoleucine when the seeds undergo abiotic stresses. This suggests that a higher content of isoleucine is required under such conditions since it is used as an osmoprotectant and a source of energy. This infers the important role of MGL under stresses.

### 4.2 The protein level of AtGML was produced in the embryo during seed development

The results obtained from plants expressing the MGL-GUS construct show that the protein of AtMGL is increased in the embryo inside the 21-26 DAF seeds. It might be that a high level of AtMGL in the embryo controls the level of SAM, which can affect methylation processes and change the chromatin structure of the seeds (An et al., 2017; Kawakatsu et al., 2017). The methylation rate increased extensively during seed development, which played a role during seed dormancy, while drastically reduced in the methylation that occurred during germination. However, a higher methylation rate beyond the required level can harm the development of the seeds (An et al., 2017; Kawakatsu et al., 2017).

The results obtained here show that the protein of AtMGL that increased in seeds is unlike those reported by Rébeillé et al. (2006). These researchers detected the protein level of AtMGL in all of the organs of WT plants, but the protein could not be detected in dry mature seeds. However, the protein of AtMGL greatly increased in the imbibed seeds (Rébeillé et al., 2006). Thus, the researchers suggest that the transcript accumulation at the dry mature stage is a prerequisite for rapid production of MGL during the imbibition process. We assume that in their case, the extraction of the seeds did not enable the antibodies to determine the MGL or other technical problems. These researchers also found that in general, no direct correlation was found between the accumulation of the transcript and the protein levels of AtMGL in the different organs of Arabidopsis WT plants, suggesting that the protein level was controlled by post-transcription regulation (Rébeillé et al., 2006). In the current study, we have an indication that this is also the case in seeds: the transcript expression level of *AtMGL* was not altered under heat stress, but the protein content significantly increased (based on the results obtained from the MGL-GUS experiment). Most probably, the activity of the MGL also increased since the level of methionine decreased and that of isoleucine increased under this stress (**Figures 2**, **3**).

### 4.3 Seeds with a lower expression level of *AtMGL* had difficulties in germinating when the seeds underwent stress

During the early stages of germination, the storage protein degrades and the AAs oxidize and degrade to produce an energy source, and/or used to form the new proteins. This occurs until the photosynthetic apparatus is fully functional (Galili et al., 2014).

The expression level of *AtMGL* was kept relatively high during the imbibition and then decreased significantly (**Figure 1**). This finding is in accordance with MGL protein accumulation during seed imbibition (Rébeillé et al., 2006). The *MGL* expression is also significantly higher in seedlings compared to other tissues and organs (Joshi and Jander, 2009). Together, these results suggest an important role of this enzyme during the germination process. Despite these studies, when the germination efficiency of seeds derived from the RNAi lines and *mgl* mutant that developed under non-stress conditions was tested, no significant changes were detected compared to their controls, EV and WT (**Figures 5**, **6**). This is similar to the results obtained in the *mgl* mutant in which the seedlings’ root lengths did not differ significantly from the WT (Joshi and Jander, 2009).

Therefore, germination efficiency was also tested in the seeds of those lines that underwent abiotic stresses during seeds development. Heat or salt stress that occur during seeds development affect the germination of the RNAi lines and the *mgl* since they had difficulties in germinating on regular agar medium compared to seeds of the EV (**Figures 5**, **6**). This suggests that the activity of AtMGL during stress is important and enables the seeds to germinate well. The low MGL function in the RNAi and in the mutant that underwent salt stress during seed development might also lead to lower yield in these plants (Schmidt et al., 2020). These seeds had significantly lower yield than their controls, suggesting a significant role of MGL during seed development and fitness. The seed weight did not change significantly between the RNAi and *mgl* lines and their controls, but the number of seeds was lower. It is also suggested that plants that have a low level of MGL prefer the seed filing process more than the number of seeds. The developmental stage in these seeds on which this decision was made is as yet unknown, but studies carried out in cereal grains suggest that it occurs several days after the anthesis. The researchers report that the occurrence of drought or heat stresses before and during anthesis reduces the number of grains due to increased seed abortion, whereas grain weight is hardly affected. In contrast, abiotic stress occurring after anthesis does not influence grain number but reduces grain size and grain weight by impeding grain filing (Sehgal et al., 2018; Schmidt et al., 2020). Since, in our case the stress continued for about 35 days, it most likely affected the anthesis in more severe ways than the control plants, suggesting a role of MGL also at this stage. Difficulties to germinate were also detected in the RNAi lines and *mgl* that were grown under regular conditions but geminated on agar supplemented with salt or mannitol. This observation strengthened the hypothesis that the MGL enzyme plays an essential role during germination under stress. Most probably under such stress conditions, a high methionine level can be toxic, or the seeds require more isoleucine. A similar assumption was also proposed for lysine. A high level of lysine in seeds resulted from mutation in its catabolic enzyme, led to a much slower rate of germination and seedling growth (Zhu and Galili, 2004). The authors suggest that a high level of lysine is toxic and should degrade during the germination process (Zhu and Galili, 2004).

The seeds of the *mgl* mutant exhibited significantly lower efficiency and germination rate compared to their control, WT, or the RNAi lines, when they germinated under salt and osmotic stresses. The sensitivity to germination under stress between the RNAi lines and *mgl* mutant might result from their nature. While the RNAi lines have a low level of MGL only during seed development and can produce MGL during the germination process, the *mgl* mutant lacks MGL in all of the organs during development. Thus, these results strengthen the assumption that newly synthesized MGL during the imbibition and germination is important for the germination process. This assumption also support from the observation that the RNAi lines that had a higher expression level of *AtMGL* than the mutant, were more tolerated to these stresses. The results also indicate that MGL produced in seeds during development plays an essential role during germination on stress conditions.

## 5 Conclusion

This study aimed at gaining more knowledge about the role of AtMGL during seed development and germination. By using isotope-labeled methionine and the *MGL* gene that fuses to GUS in plants exposed to stress conditions in a short-term experiment, we can conclude that AtMGL becomes an active enzyme when plants and their seeds cope with abiotic stresses such as heat, osmotic or salt stress. The finding that this enzyme is active during abiotic stress in seeds suggests that under such conditions, MGL activity is required to produce isoleucine. Isoleucine plays a significant role in stress as an osmoprotectant and a source of energy. The experiment that used the MGL-GUS revealed that the protein of AtMGL is translated in the embryo of the seeds, but its level significantly increased under heat stresses.

RNAi::AtMGL lines and the *mgl* mutant that develop during heat or salt stress have difficulties in germinating under non-stress conditions. This suggests an essential role of MGL during seed development, which enables the seeds to germinate well. Also, the RNAi seeds that develop under regular conditions but germinate under salt and osmotic conditions exhibit low germination efficiency, indicating that AtMGL is active during seeds maturation and is essential for the early stages of germination. The differences in germination efficiency between the RNAi lines and *mgl* mutant suggest an important role of the newly synthesized MGL during the germination process.

From a biotechnology point of view, increasing the contents of methionine and isoleucine, two essential amino acids in seeds and other edible plant organs, has been a goal for breeding and agricultural biotechnology. The observation that MGL makes a significant contribution to methionine degradation, particularly in seeds, proposes practical applications for silencing the expression of *MGL* in the seeds of crop plants and other edible organs to increase the abundance of methionine (Amir, 2010; Amir et al., 2012; Huang et al., 2014; Kumar and Jander, 2017). Also, a reduced expression level of *MGL* in seeds can trigger the synthesis of isoleucine from threonine deaminase (Joshi and Jander, 2009). However, our results show that under non-stress conditions, the level of methionine does not increase in the seeds of the RNAi lines. Moreover, the activity of MGL is essential for seed development and during germination, and these roles can be seen especially when the seeds undergo stress during their development and germination processes. Therefore, such a strategy to increase methionine levels in seeds using the RNAi method is not worthwhile. However, since a higher level of methionine was observed in the seeds of the *mgl* mutant, it is possible to carry out a Crispr-Cas method to knock out the *MGL*, which could lead to a higher level of methionine. If this strategy will be chosen, a further study would be required to determine the effect on the ability of these plants to cope with stresses (including yield and germination rate).

## Data availability statement

The original contributions presented in the study are included in the article/**Supplementary Material**. Further inquiries can be directed to the corresponding author.

## Author contributions

YH and RA: experimental design. YH, OS, ON, and MF: conducting the experiments. YH, OS, ON, and MF: data analysis. RA: manuscript preparation. All authors contributed to the article and approved the submitted version.
