# Supplementary material for: Elucidating the importance of the catabolic enzyme, methionine-gamma-lyase, in stresses during Arabidopsis seed development and germination
Source: Front Plant Sci. 2023 Jun 6;14:1143021. doi: 10.3389/fpls.2023.1143021 (PMC10280021; doi:10.3389/fpls.2023.1143021)
Supplement: Supplementary file 1 [file Presentation_1.pptx]

## Slide 1
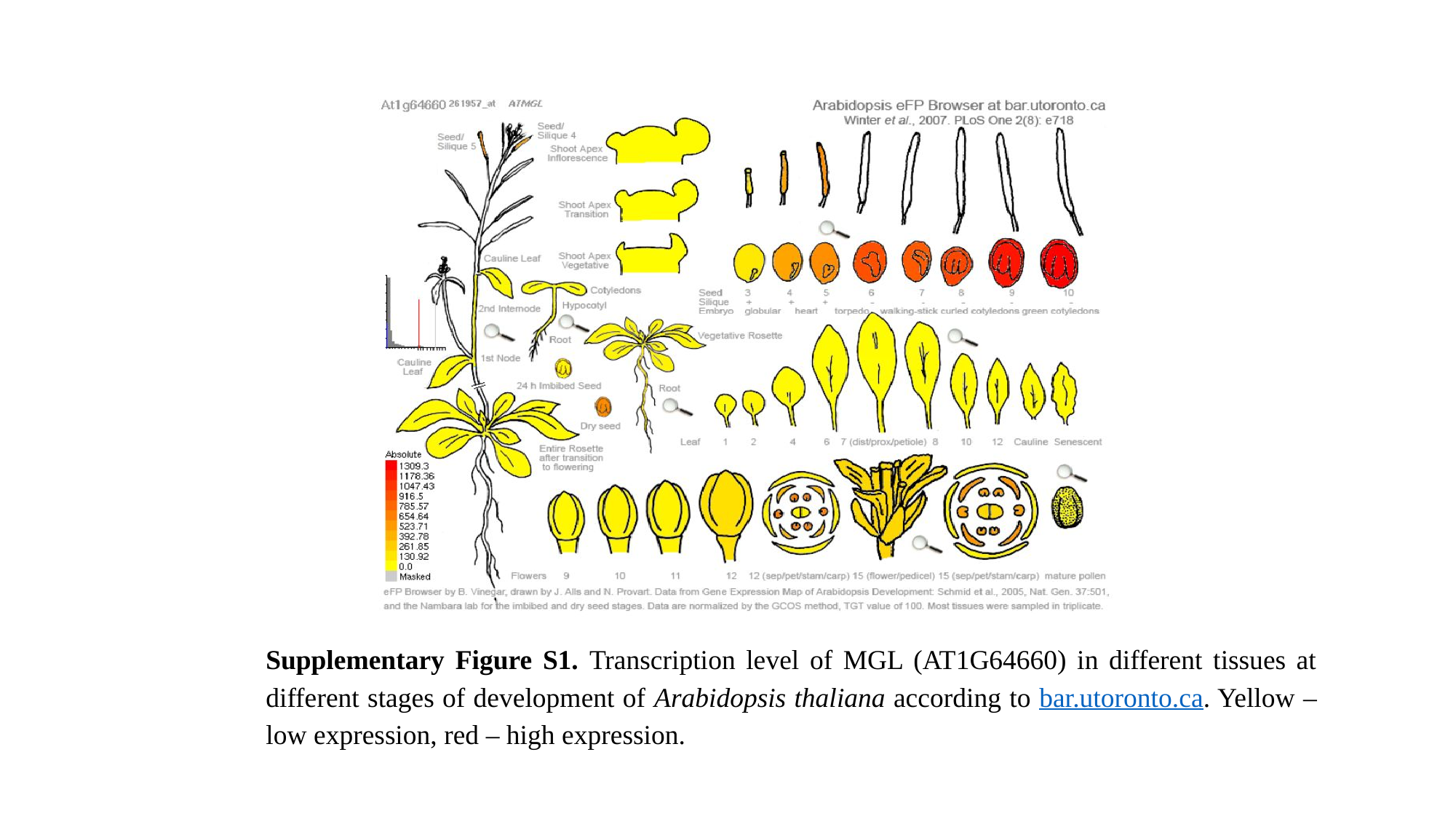

Supplementary Figure S1. Transcription level of MGL (AT1G64660) in different tissues at different stages of development of Arabidopsis thaliana according to bar.utoronto.ca. Yellow – low expression, red – high expression.

## Slide 2
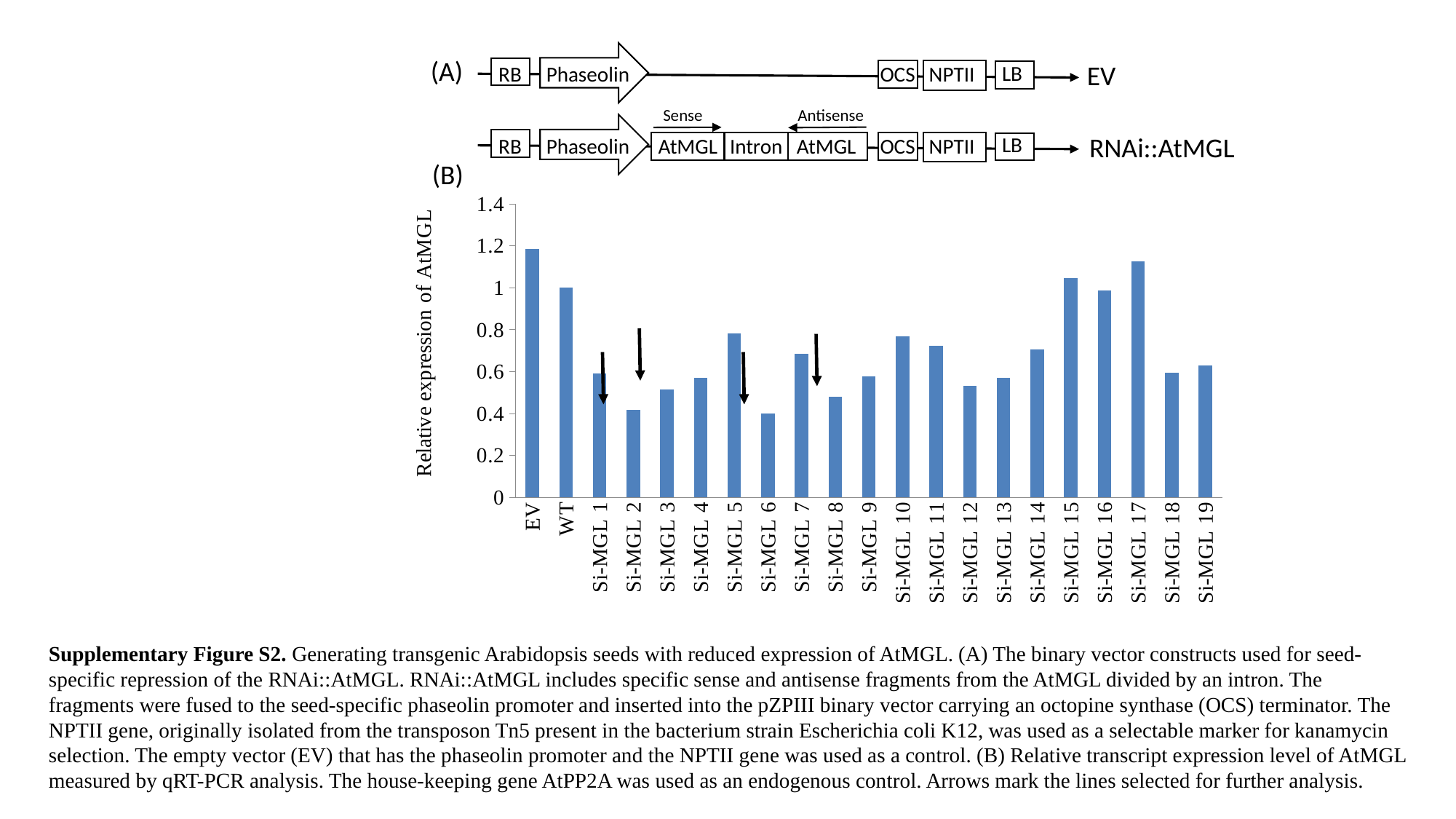

LB
RB
Phaseolin
OCS
NPTII
(A)
EV
Sense
Antisense
LB
RB
Phaseolin
AtMGL
Intron
AtMGL
OCS
NPTII
RNAi::AtMGL
(B)
### Chart
| Category | |
|---|---|
| EV | 1.1865485069297947 |
| WT | 1.0 |
| Si-MGL 1 | 0.59 |
| Si-MGL 2 | 0.4172013251865419 |
| Si-MGL 3 | 0.5138185639389663 |
| Si-MGL 4 | 0.57 |
| Si-MGL 5 | 0.7815285455100633 |
| Si-MGL 6 | 0.4 |
| Si-MGL 7 | 0.6868660025268906 |
| Si-MGL 8 | 0.48 |
| Si-MGL 9 | 0.5765382623330838 |
| Si-MGL 10 | 0.7689668067012665 |
| Si-MGL 11 | 0.7249598640994923 |
| Si-MGL 12 | 0.530818804207316 |
| Si-MGL 13 | 0.5702317283235343 |
| Si-MGL 14 | 0.7050027903850623 |
| Si-MGL 15 | 1.0481636426674836 |
| Si-MGL 16 | 0.9881043672780326 |
| Si-MGL 17 | 1.126887548848715 |
| Si-MGL 18 | 0.59405230076037 |
| Si-MGL 19 | 0.6294255745551572 |Supplementary Figure S2. Generating transgenic Arabidopsis seeds with reduced expression of AtMGL. (A) The binary vector constructs used for seed-specific repression of the RNAi::AtMGL. RNAi::AtMGL includes specific sense and antisense fragments from the AtMGL divided by an intron. The fragments were fused to the seed-specific phaseolin promoter and inserted into the pZPIII binary vector carrying an octopine synthase (OCS) terminator. The NPTII gene, originally isolated from the transposon Tn5 present in the bacterium strain Escherichia coli K12, was used as a selectable marker for kanamycin selection. The empty vector (EV) that has the phaseolin promoter and the NPTII gene was used as a control. (B) Relative transcript expression level of AtMGL measured by qRT-PCR analysis. The house-keeping gene AtPP2A was used as an endogenous control. Arrows mark the lines selected for further analysis.

## Slide 3
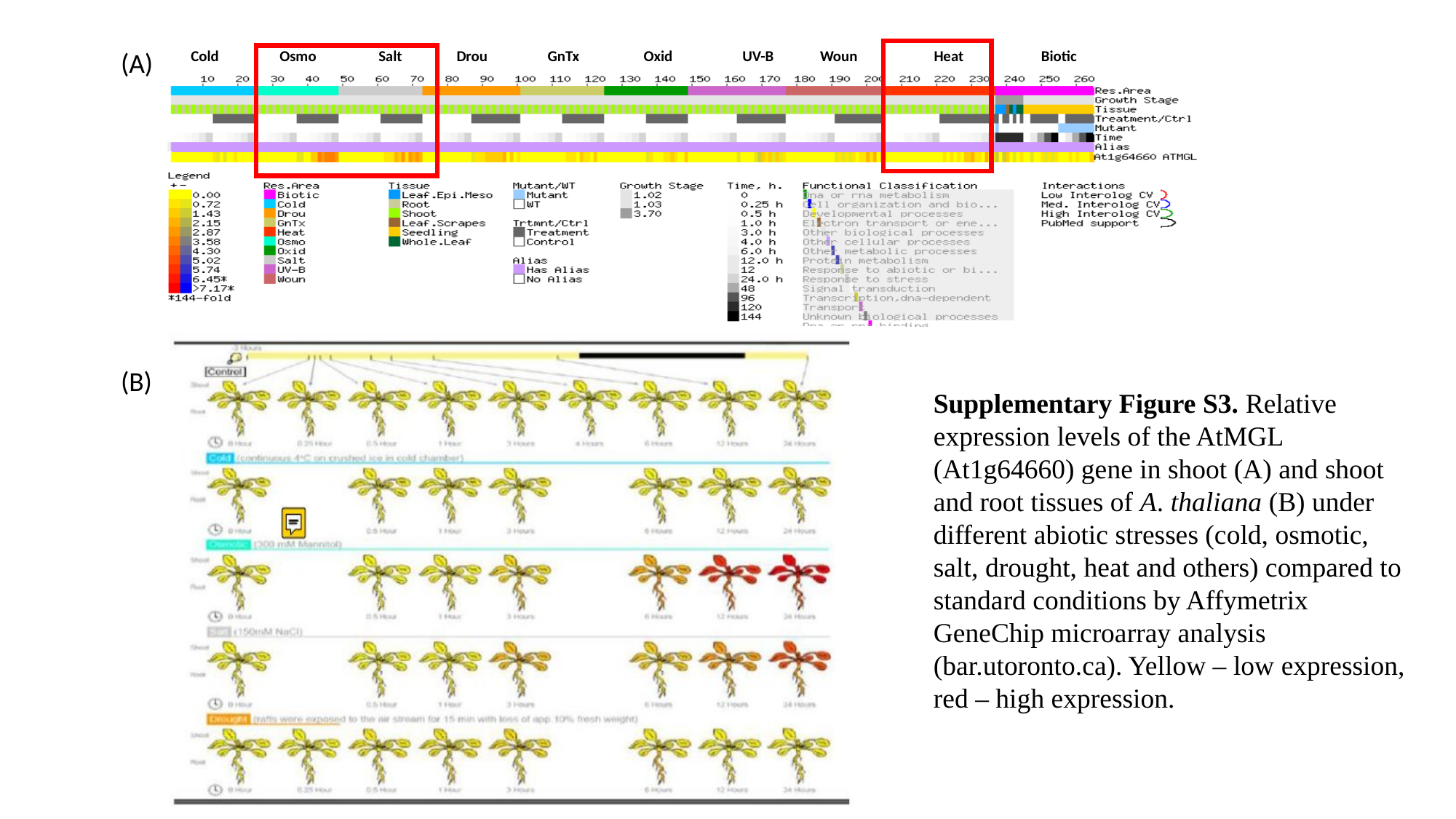

(A)
Biotic
Woun
UV-B
Oxid
Cold
Heat
Salt
Osmo
Drou
GnTx
(B)
Supplementary Figure S3. Relative expression levels of the AtMGL (At1g64660) gene in shoot (A) and shoot and root tissues of A. thaliana (B) under different abiotic stresses (cold, osmotic, salt, drought, heat and others) compared to standard conditions by Affymetrix GeneChip microarray analysis (bar.utoronto.ca). Yellow – low expression, red – high expression.

## Slide 4
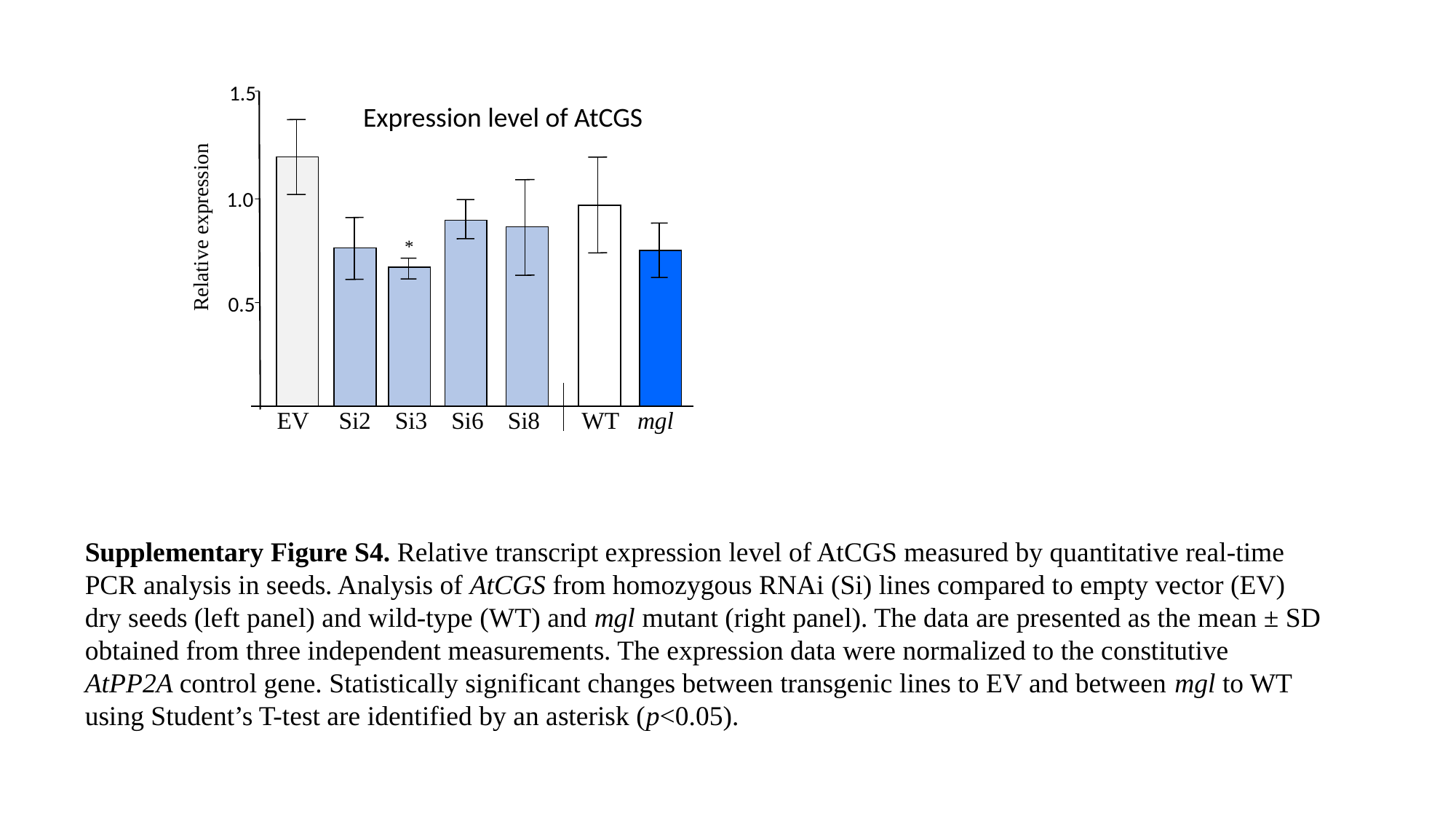

1.5
1.0
Relative expression
*
0.5
Expression level of AtCGS
EV Si2 Si3 Si6 Si8 WT mgl
Supplementary Figure S4. Relative transcript expression level of AtCGS measured by quantitative real-time PCR analysis in seeds. Analysis of AtCGS from homozygous RNAi (Si) lines compared to empty vector (EV) dry seeds (left panel) and wild-type (WT) and mgl mutant (right panel). The data are presented as the mean ± SD obtained from three independent measurements. The expression data were normalized to the constitutive AtPP2A control gene. Statistically significant changes between transgenic lines to EV and between mgl to WT using Student’s T-test are identified by an asterisk (p<0.05).

## Slide 5
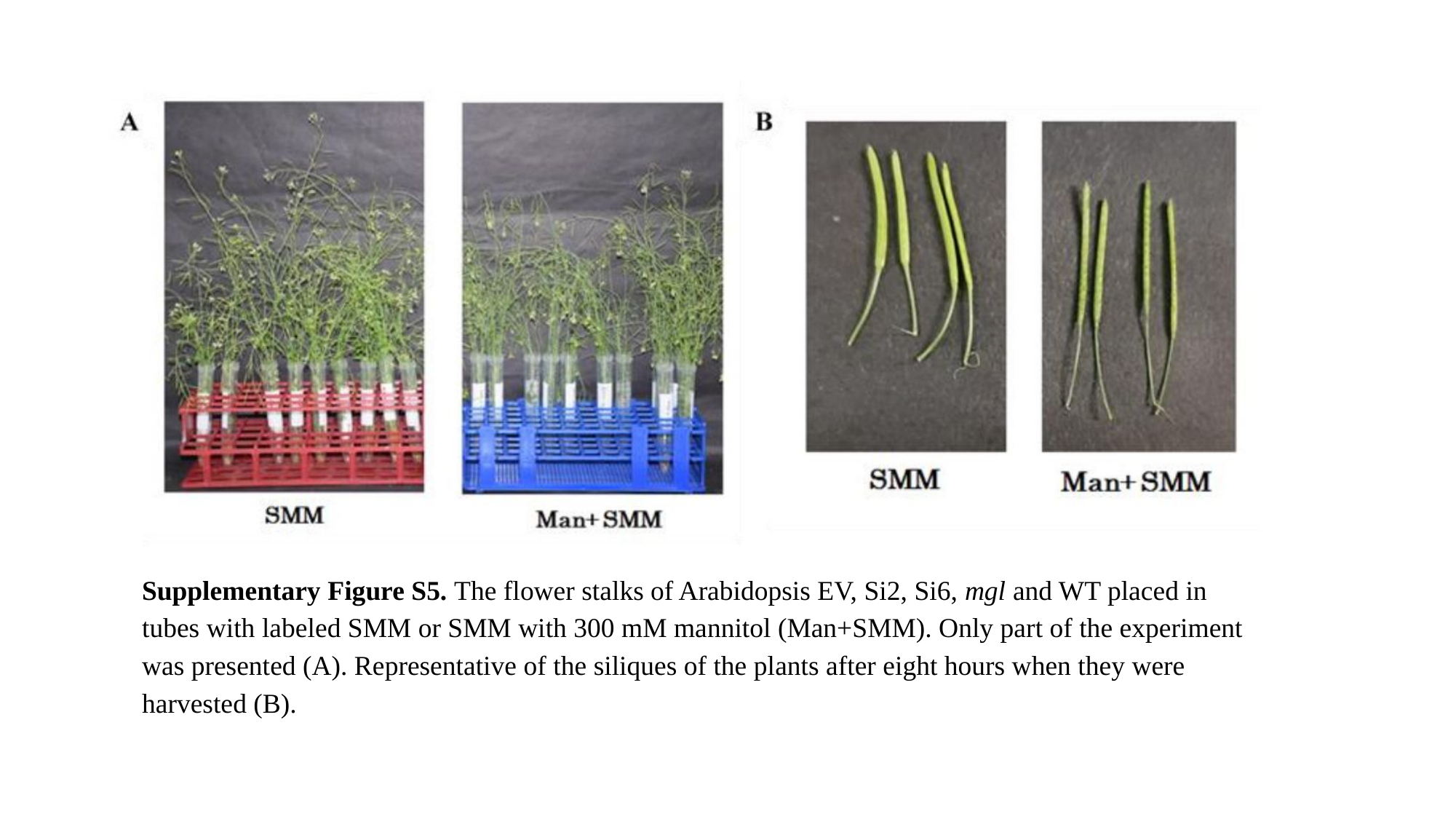

Supplementary Figure S5. The flower stalks of Arabidopsis EV, Si2, Si6, mgl and WT placed in tubes with labeled SMM or SMM with 300 mM mannitol (Man+SMM). Only part of the experiment was presented (A). Representative of the siliques of the plants after eight hours when they were harvested (B).

## Slide 6
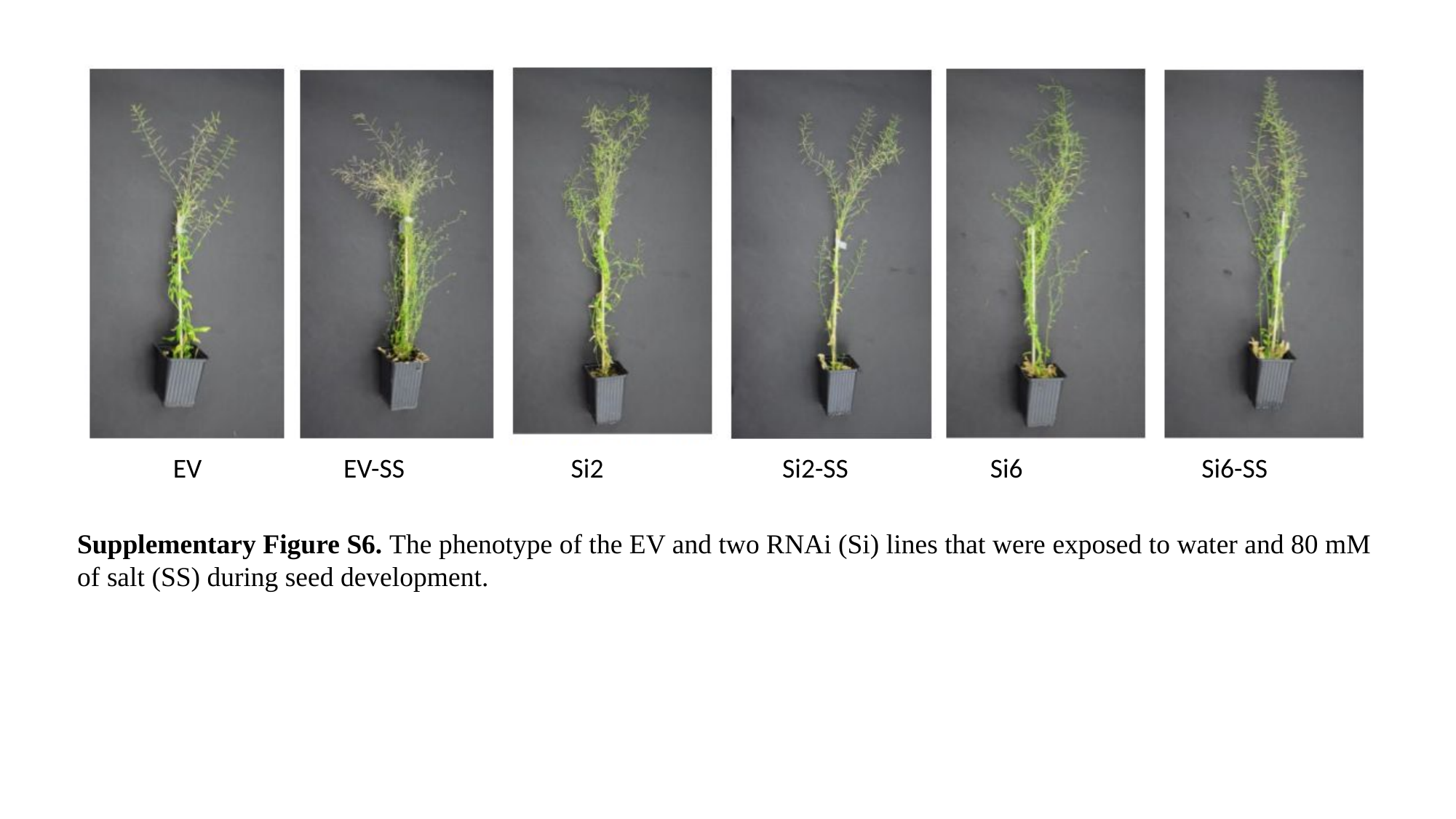

EV EV-SS Si2 Si2-SS Si6 Si6-SS
Supplementary Figure S6. The phenotype of the EV and two RNAi (Si) lines that were exposed to water and 80 mM of salt (SS) during seed development.
